# A quantitative model for dermal infection and oedema in BALB/c mice pinna

**DOI:** 10.1186/s12866-016-0907-0

**Published:** 2016-12-12

**Authors:** Erika Nahomy Marino-Marmolejo, Flor Yohana Flores-Hernández, Mario Alberto Flores-Valdez, Luis Felipe García-Morales, Ana Cecilia González-Villegas, Jorge Bravo-Madrigal

**Affiliations:** 1 Centro de Investigación y Asistencia en Tecnología y Diseño del Estado de Jalisco A.C. Biotecnología Médica y Farmacéutica, Av. Normalistas No. 800. Colinas de la Normal, C. P. 44270 Guadalajara, Jalisco México; 2 OPKO México, Pharmacos Exakta S.A. de C.V. Laboratorio de Investigación y Desarrollo, Av. Niño Obrero No. 651. Chapalita Sur, C. P. 45040 Zapopan, Jalisco México

**Keywords:** Animal model, Dermal infection, Oedema, *Streptococcus pyogenes*

## Abstract

**Background:**

Pharmaceutical industry demands innovation for developing new molecules to improve effectiveness and safety of therapeutic medicines. Preclinical assays are the first tests performed to evaluate new therapeutic molecules using animal models. Currently, there are several models for evaluation of treatments, for dermal oedema or infection. However, the most common or usual way is to induce the inflammation with chemical substances instead of infectious agents. On the other hand, this kind of models require the implementation of histological techniques and the interpretation of pathologies to verify the effectiveness of the therapy under assessment.

This work was focused on developing a quantitative model of infection and oedema in mouse pinna. The infection was achieved with a strain of *Streptococcus pyogenes* that was inoculated in an injury induced at the auricle of BALB/c mice, the induced oedema was recorded by measuring the ear thickness with a digital micrometer and histopathological analysis was performed to verify the damage. The presence of *S. pyogenes* at the infection site was determined every day by culture.

**Results:**

Our results showed that *S. pyogenes* can infect the mouse pinna and that it can be recovered at least for up to 4 days from the infected site; we also found that *S. pyogenes* can induce a bigger oedema than the PBS-treated control for at least 7 days; our results were validated with an antibacterial and anti-inflammatory formulation made with ciprofloxacin and hydrocortisone.

**Conclusions:**

The model we developed led us to emulate a dermal infection and allowed us to objectively evaluate the increase or decrease of the oedema by measuring the thickness of the ear pinna, and to determine the presence of the pathogen in the infection site. We consider that the model could be useful for assessment of new anti-inflammatory or antibacterial therapies for dermal infections.

## Background

The skin is our major barrier against biological threats such as bacterial pathogens. An infection in the skin usually begins with a damage or injury in the epithelium. *Pseudomonas aeruginosa* and *Staphylococcus aureus* are frequently involved in dermal infections, when the infection is localized n the external auditory canal, it is commonly defined as external otitis [1, 2]. *Streptoccocus pyogenes* is a specific pathogen that is able to cause severe skin infections after a previous dermal lesion [3].

There are several therapies for treating cutaneous infections, some of them are delivered systemically or applied locally; the last are topical substances which can have anti-inflammatory and/or antibacterial effects [2]. Nowadays there are a plethora of new medical formulations that have been analyzed to evaluate their therapeutic qualities against dermal diseases [4–6]. For the assessment of those drugs is necessary to include evaluations of both antibacterial and/or anti-inflammatory effects, and these should be tested in a model that could display most of the signals seen in dermal illness, such as oedema, erythema, exudate and the etiological agent in the site of infection. Several animal models, developed to quantitatively evaluate the anti-inflammatory effect of topical drugs, induce a dermal oedema usually localized at the pinna or on external auditive canal through mechanical lesions [7]. These models require chemical substances such as 12-O-tetradecanoylphorbol-13-acetate (TPA) [8–10], cantharidine [11], capsaicin [12, 13] or Zymosan [14] to induce the oedema at the pinna. Although there have been developed models of ear or dermal infection with *Pseudomonas*, *Candida* [15], *Streptococcus pyogenes* or *Staphylococcus aureus* [16] and those models allow to analyze the infectious process, it is not easy to quantitatively measure the induced oedema. Because it is common that several treatments or formulation could display both anti-inflammatory and antibacterial effects [17] therefore, it would be useful to analyze both effects in the same model.

The innovations in the development of new antibacterial or anti-inflammatory drugs, or improving of the current formulations or vehicles for their delivery, require objective methods for their assessment. In fact, it should be useful, that procedures not based in the experience or human criteria exist. In this sense, it is convenient to develop quantitative and robust methods, that could be carried out in a blind way, and whose results would be reproducible. Likewise, the agent responsible for inducing the inflammation or oedema, should be a biological agent able to infect the host, because the infection involves a more complex immunological response than those induced by chemicals substances.

## Methods

The aim of this work was to develop an animal model of dermal infection in mouse pinna that emulate the signals found in a typical cutaneous infection, that could be reproducible and quantitative, for testing antibacterial and/or anti-inflammatory therapies. Our results were verified by using the standard histopathological techniques for assessment of tissue damage. The model presented here involves the use of *Streptococcus pyogenes* as inductor of skin infection and oedema on mouse auricle. Although this bacteria is not a main skin pathogen, some strains are able to cause secondary skin infections such as impetigo [3]. On the other hand, it has been already evaluated that *S. pyogenes* can infect skin in mice [16, 18, 19].

### Animals

Female BALB/c mice, 13- to 15-week-old (Harlan, Mexico) were maintained in an environmental controlled room (24 °C, artificial lightning with circadian cycle of 12 h, ten air changes per h), and were allowed free access (*ad libitum*) to standard food for mice (Harlan, Mexico) and purified water; mice were acclimated 3 weeks before any procedure. All experiments were performed in aseptic areas, just before beginning the experiments; each mouse was kept in individual cages with sterilized wood shavings (Harlan, Mexico). Ethylic ether vapors (Sigma Aldrich Co., St. Louis, Mo) were used as anesthetic during procedures of inoculation and measuring of ear thickness. We carried out a strict surveillance to avoid animal suffering. In the same way, all experiments were performed in accordance with the government laws for Laboratory animal care and use guidelines [20].

### Strain

*Streptococus pyogenes* was the specie chosen for the model; the strain was previously isolated from a 9 years old child with impetigo. The strain was cultured in Blood Agar (Becton and Dickinson, Mexico Ref 200150) at 36.5 °C; after 18 h we inoculated three colonies in one BALB/c mouse auricle, the pinna was pierced with three 23-gauge hypodermic sterile needles arranged in triangular shape. The mouse was maintained in a sanitized cage during 3 days. After that, *S. pyogenes* was isolated in blood agar from the infection site; the new strain was labeled as R2. In order to induce adaptation to mice, the strain R2 was inoculated again in another mouse, but previously the strain was cultured 6 h at 37 °C in Minimal Essential Medium (Sigma Aldrich Co., St. Louis, Mo, Ref M0643) supplemented with 10% Fetal Bovine Serum (ATCC, Manassas, VA, Ref 30–2020) (MEM-FBS). The bacterial culture was centrifuged for 10 min at 3000 xg, the supernatant was discarded and the bacterial pellet was resuspended and adjusted at 1 × 10^10^ CFU/ml (previously, a nephelometric analysis and viable count was carried out to quantify the CFU/ml according to its absorbance at 600 nm). 10 μl of this bacterial suspension was inoculated on the mouse pinna following conditions previously described above. The aim of this experiment was to recover the most adaptable *S. pyogenes* strain. A bacterial isolation was carried out every day to recovery the most able strain to remain in the infection site, and the strain recovered was labeled as R3. We repeated this procedure 5 times more and we obtained the R8 strain, which could remain 4 to 8 days in the mouse pinna. This strain was sent to ARS Culture and Patent Culture Collections in where is available, as *Streptococcus pyogenes* B-50879.

### Animal inoculation

B-50879 strain was cultured in (MEM-FBS) following conditions previously described above, 3 h before inoculation we recorded the weight of mice, and the auricles of each mouse were sanitized with 0.1% benzalkonium chloride. Mice were exposed to Ethylic ether vapor 15 to 20 min into a container which had a sterilized filter and dry cotton to avoid cross-contamination and direct contact of the solvent with the mouse. Just before inoculation, the ear thickness was measured 5 times by using a sanitized digital micrometer (Fowler, Newton, MA) that has an accuracy of 0.01 mm, after that, the mouse pinna was sanitized with 70% ethylic alcohol. The site of inoculation was injured with 3 punctures and immediately was inoculated with 10 μl of 1 × 10^8^ CFU of the B-50879 strain adjusted in phosphate buffer saline pH 7.2 (PBS) (Gibco-Invitrogen, Grand Island, NY) as previously described, after inoculation the mouse was returned to its cage. Two areas in the mouse ear were tested for inoculation: The center of the pinna, that was pierced as previously described, with a punch made with 3 needles arranged in a triangular shape and spaced 3 mm and the edge of the pinna that was also pierced with a 23-gauge hypodermic needle in three sites around the auricle. After every measure or inoculation, all instrumental was sanitized with 70% ethanol, to avoid cross-contamination.

Every day after inoculation, mice were weighted and a swab (Becton Dickinson, Franklin Lakes, NJ, Ref 220131) from the inoculation site was cultured in blood agar to verify if *S. pyogenes* were present, also the induced oedema was recorded by measuring five times the ear thickness with a digital micrometer as previously described. Phenotypic test such as Pirrolidonyl arylamidase (PIR) test (Oxoid, Hampshire, United Kingdom) and sensibility to Bacitracin (0.04 U. Oxoid, Hampshire, United Kingdom) were performed to confirm the identity of *S. pyogenes.*

### Morphological and histopathological analysis

Mice from each group at 2 to 6 days post-inoculation were euthanized by cervical dislocation, and biopsies from each auricle were collected, fixed in 10% Formalin-PBS, and paraffin-embedded. The tissues were sliced at a thickness of 10 μm by using a microtome (Leica, Wetzlar, Germany), Gram and hematoxylin-eosin (HE) stains were performed, and were observed in light microscopy (Leica, Wetzlar, Germany). All microphotographs were captured by using a digital camera and analyzed with the software Infinity Analyze® (Lumenera Corporation, Ottawa, Canada). Leucocyte infiltration in infected pinna and PBS-treated pinna were semi quantified in the HE preparations, total inflammatory cells were counted from 8 fields at 50x.

### Hematological assays

Blood samples from mice in each group were collected in the presence of EDTA, before and 24 h post-inoculation. 10 μl of blood were diluted 1:40 in Turk reagent (Merck, Darmstadt, Germany) and total leukocytes were counted by light microscopy by using the hemocytometer (Hausser Scientific, Horsham, PA). Differential count of leukocytes was performed by staining blood slides with Wright’s reagent (Sigma Aldrich Co., St. Louis, Mo, Ref WS32) according to manufacturer’s instructions, and 200 cells were counted under light microscopy so as to quantify the number of neutrophils.

### Protocol of validation

A commercial treatment made with Ciprofloxacin 2 mg/ml, Hydrocortisone 10 mg/ml and Benzocaine 20 mg/ml (Sodrimax®, PiSA Farmaceutica, Mexico) was used as antimicrobial and anti-inflammatory standard, for comparison we used a sterilized glycerol 50% in PBS as placebo. All treatments were maintained and delivered in a double-blind way. Twelve mice were inoculated with *S. pyogenes* B-50879 in both pinna as previously mentioned. Treatments were evaluated in two ways: as curative, in where both formulations were applied 24 after the inoculation; and as preventive way, in where the treatments were administered 1 h after inoculation. 10 ul from each solution was delivered on the pinna surface every 8 h during 8 days, every day in the morning before the treatment, was taken a swab from the pinna and was measured the ear thickness. When the protocol finished, animals were euthanized, following the government laws for Laboratory animal care and use guidelines [20]. Bacterial presence and absence was compared in the infected pinna treated with antibacterial o placebo treatments, furthermore the oedema inhibition was evaluated with data collected from the ear thickness measurements during the protocol and compared with the placebo.

### Statistical analysis

Results are expressed as mean of the group ± standard error of the media (SEM), paired t student test was used for intragroup comparison (dependent variable influenced by the individual, for example left vs right pinna or placebo vs treatment). Non-paired t student test was applied when two independent groups were compared (Group treated with *S. pyogenes* vs group control-PBS-treated). Statistical analysis was performed every day between two groups considering *p* <0.05 as significant. Using a variant of the surviving test, in which the time of permanence of *S. pyogenes* at the infection site was analyzed, compared the permanence of *S. pyogenes*, Wilcoxon test was used to infer statistical difference considering *p* <0.05 as significant. *x* ^2^ test was used to evaluate differences between placebo and antibacterial treatment with a 2 × 2 contingency table according to the Mantel Haenszel procedure, considering *p* <0.05 as significant. All statistical tests were carried out in the software STATgraphics Centurion XVI (StatPoint Technologies, Warrenton, VA).

## Results

Animal models are useful for research in medical sciences. They aim to emulate the classical signs of the illness; for models of dermic infection the evidence of inflammation and infection should be demonstrated. *S. pyogenes* B-50879 was used in our method as an infectious agent able to cause a quantifiable oedema. We performed morphologic and histopathological analysis as well as microbiologic assays to ensure the signals of dermic infection. On the other hand, quantitative analysis of eodema was performed, to assess its reproducibility and utility, to differentiate inflammation caused by the procedure, from the inflammation caused by the dermic infection.

### Histopathological and morphological findings

**Fig. 1:**
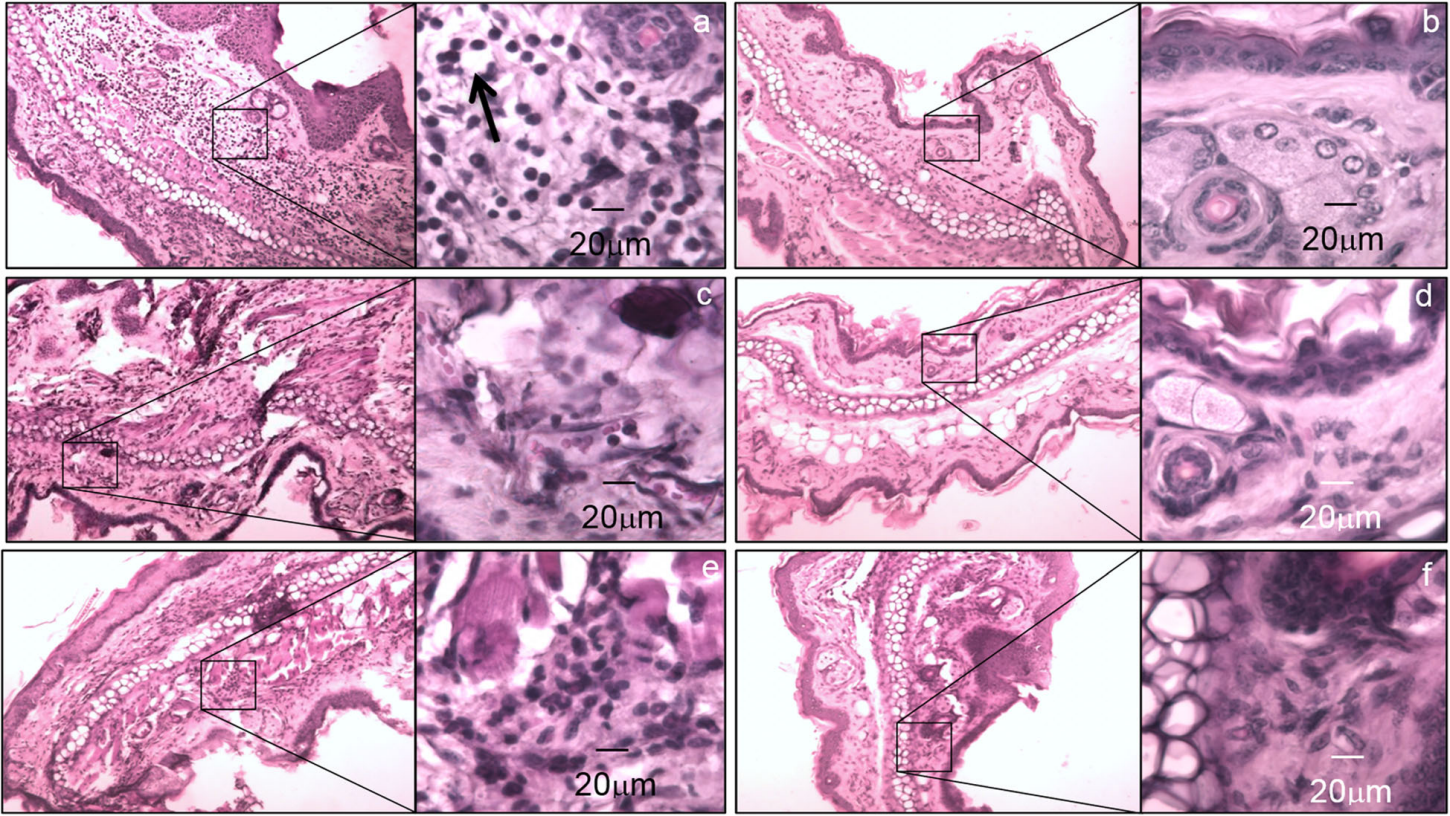
Dermal oedema induced by *S. pyogenes*. Microphotographs of BALB/c mouse pinna after inoculation in a previous injured area. The pictures were captured with an objective of 10x and the squares show the 500× magnification. **a**, **c** and **e**) Mouse pinna after 2, 4 and 6 days post inoculation with *S. pyogenes* respectively, the arrow shows white areas that might be oedema into the tissue. **b**, **d** and **f**) PBS-treated control at 2, 4 and 6 days after inoculation respectively

*S. pyogenes* B-50879 could induce an inflammatory response, when it was inoculated on an injured mouse pinna. Polymorphonuclear cells were abundant in tissues infected, we also observed hyperplasia in the spinosum stratum and important oedema into the dermis. Although the process needed for the infection involve inducing damage on the pinna, we found that PBS-treated controls did not show the same number of inflammatory cells or the same magnitude of oedema induced by the bacterium (Fig. 1). Total Inflammatory cells from tissues at 48 h were semi quantified in order to determine the leukocyte infiltration on histological preparations, we found 4 times more inflammatory cells (an average of 78 vs. 18 cell/40X field) into the tissues infected with *S. pyogenes* in comparison with PBS-treated control, the difference was statistically significant (Non-paired t student, *P* = 0.0026).

**Fig. 2:**
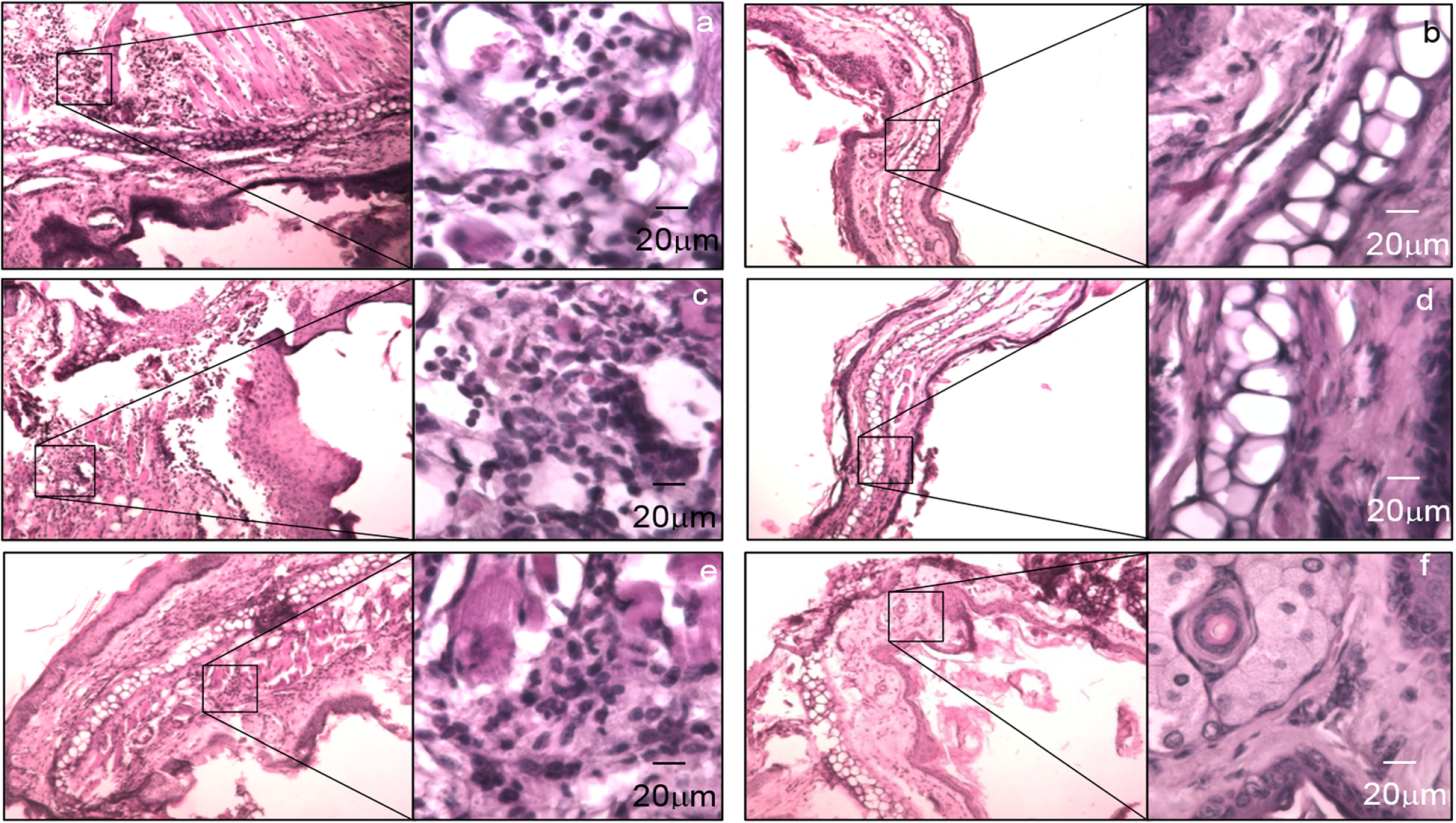
Effect of the physical injury on the mouse pinna in the development of oedema caused by the inoculation of *S. pyogenes*. The photographs were captured with an objective of 10x and the squares show the 500x magnification. **a**, **c** and **e**) Histological changes observed in the pinna at 2, 4 and 6 days post inoculation respectively, the infection was carried out on a previous injured mouse auricle. **b**, **d** and **f**) No changes were observed in the pinna at 2, 4 and 6 days post inoculation when the bacterium was delivered in the skin of the auricle without any injury

When we inoculated *S.pyogenes* without previously-induced damage, we found neither inflammatory cells nor oedema into the tissue, showing similar results as the control non-inoculated; (Fig. 2). All histopathological findings agreed with signals observed in dermal infections.

**Fig. 3:**
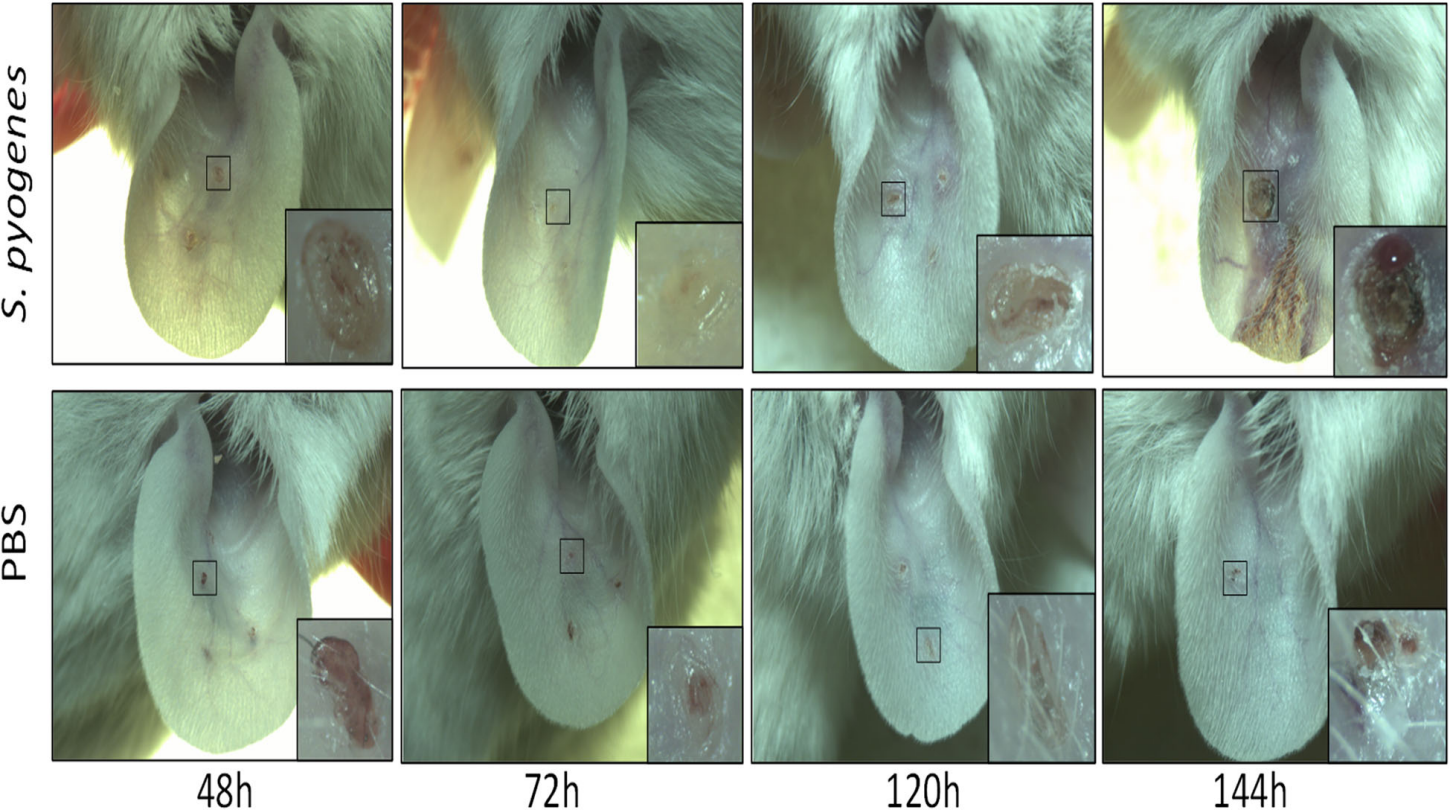
Macroscopic effect observed in auricle infected with the *S. pyogenes* strain B-50879. Auricles from mice inoculated over a small damage, the squares show a 30x magnification of the indicated area. *S. pyogenes* could cause exudate, erythema, eschars and delivery of purulent liquid. The PBS-treated control only showed a small erythema all-around the injury previously induced; finally, the damage could heal faster than ones inoculated with the bacterium

Mice pinna infected with *S. pyogenes* showed an increase in its thickness, erythema, yellow-colored crusts and delivery of purulent liquid. Most of the lesions healed during the first 6 days; however, a 5^th^ part delayed its healing until 8 to 10 days, (Fig. 3). PBS-treated control showed lower erythema, although we found crusts in the site of inoculation, it was smaller and they healed in an average of 5 days, also we did not observe purulent liquid in any of these animals. Some infection can affect the weight of the host; however, we did not find difference between animals infected and controls (data not shown).

We found that 2 of 54 mice (in a total of five experiments: *n* = 10, *n* = 14, *n* = 16, *n* = 7, *n* = 7) developed a heightened inflammatory response with the presence of a crust that prevented proper measurement of the ear thickness (Fig. 3, 144 h). To avoid affecting the accuracy of the method we decided to disregard the data from these animals.

**Fig. 4:**
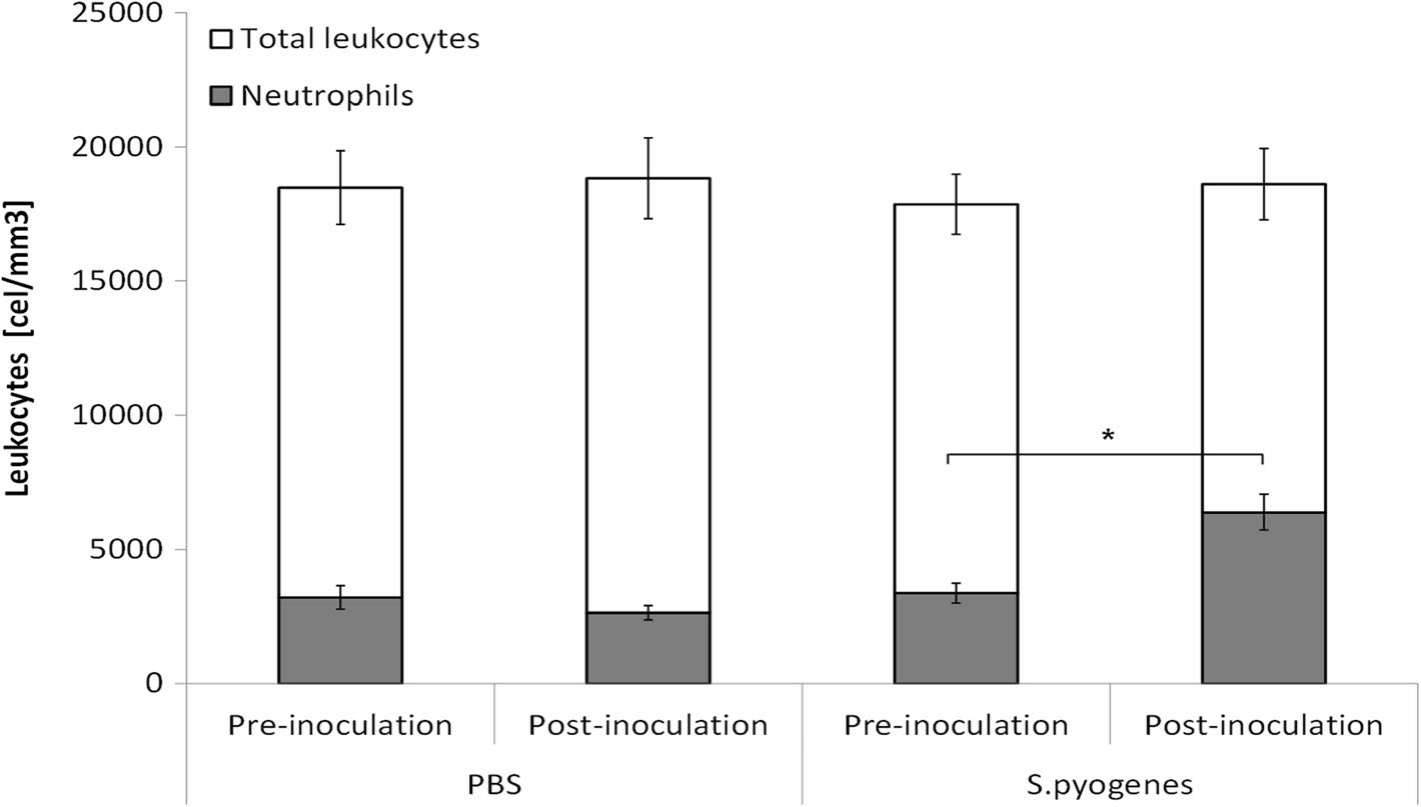
Hematological effect of inoculation of *S. pyogenes* on mouse pinna. A significate increase of neutrophils was recorded 1 day after the inoculation of *S. pyogenes* on damaged mouse pinna (*n* = 24, *p* = 0.0011; paired t student test). The PBS-Treated control did not show differences after the inoculation, (*n* = 14, *p* = 0.33; paired t student test)

Infectious diseases often induce an increase in leukocytes from the peripheral blood; the infection conducted in mouse pinna showed that there was only a significant increase of neutrophils 24 h after inoculation (*P* = 0.0016), but the total counts of leukocytes were not significantly different when we compared results before and after inoculation. PBS-treated control group did not show difference in neutrophils or in total leukocytes. (*P* = 0.33) (Fig. 4).

### Bacterial clearance at the site of infection

**Fig. 5:**
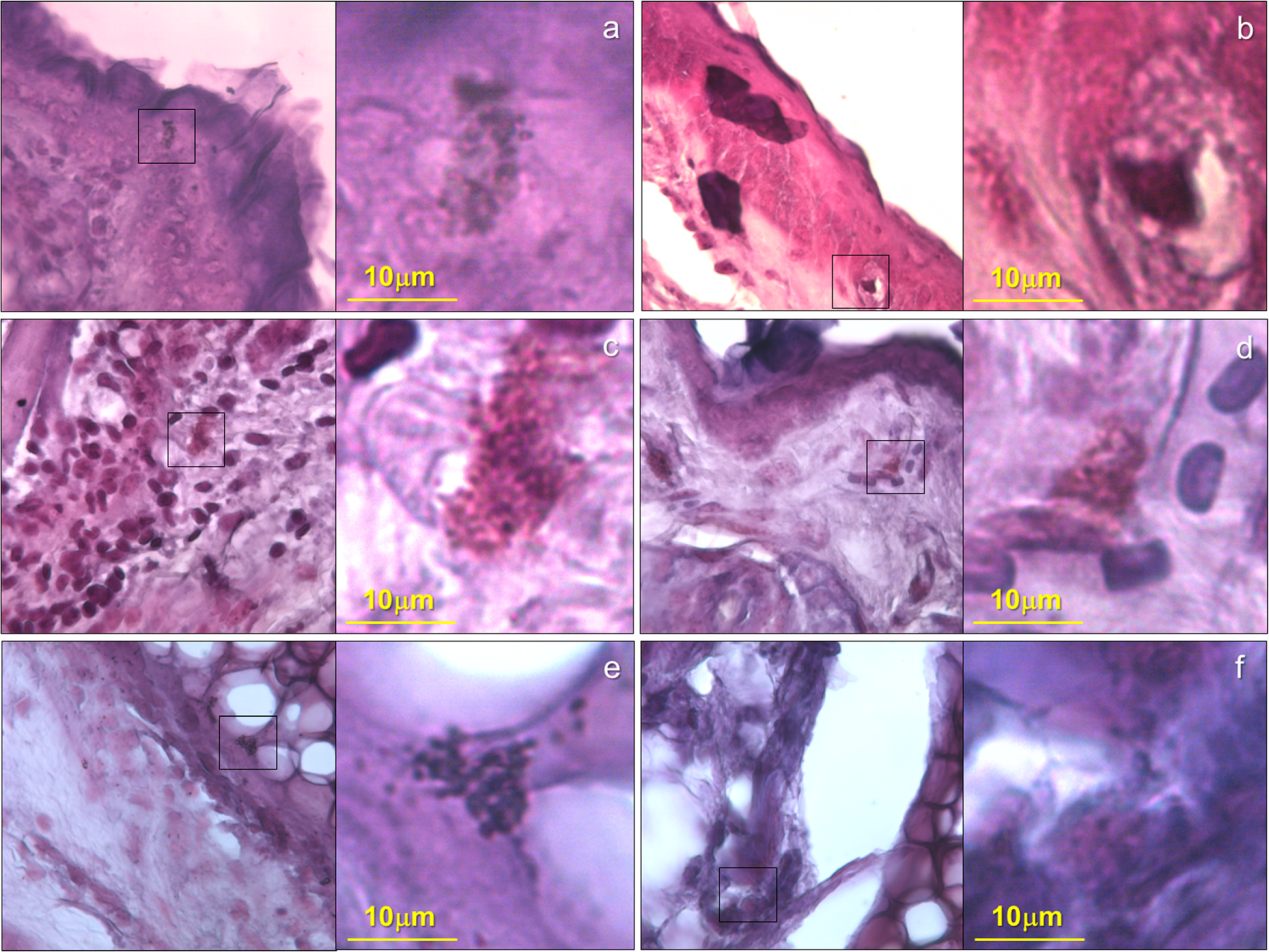
Gram stain that shows cocci into the mouse pinna previously infected with *S. pyogenes* strain B-50879. The images are representative fields at 40x of the tissue from pinna of mice infected or PBS-treated after previous damage and stained with GRAM. The squares were amplified with the immersion oil objective to show a 1000x magnification. **a**, **c** and **e** are tissue infected with *S. pyogenes* at 48 h, 96 h and 144 h post inoculation. **b**, **d** and **f** are tissue treated with PBS at 48 h, 96 h and 144 h respectively

We observed that *S. pyogenes* could survive near to the infection site. Sections of infected tissue stained by Gram showed bacterial cocci that resemble streptococcus, the bacteria were visible into the tissue until 6 days post-inoculation, PBS-treated controls did not show any bacteria into the tissue (Fig. 5).

**Fig. 6:**
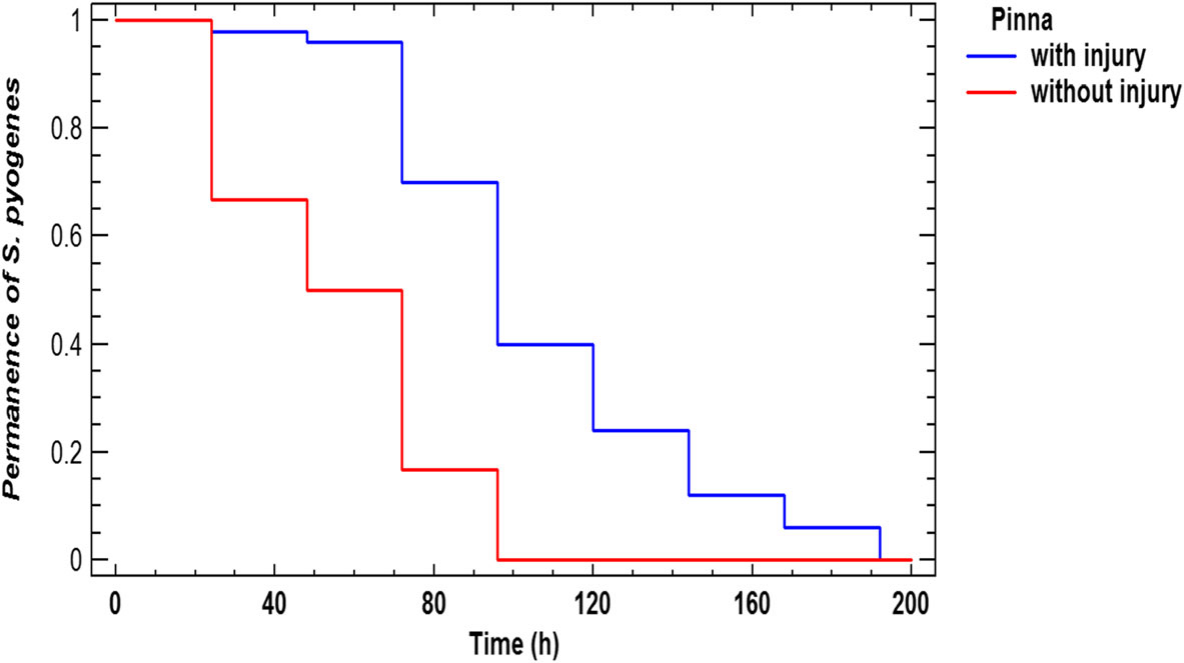
Permanence of *S. pyogenes* in the pinna. The image represents the likely of recovery *S. pyogenes* from a mouse pinna after the process of inoculation, a previous damage on the mouse pinna (*n* = 50), let us to recover bacteria after longer time than pinna without injury (*n* = 12). Wilcoxon test *P* <0.00001

Swabs obtained from the site of inoculation were cultured; *S. pyogenes* was isolated from infected auricles but not from controls. Mice could clear the bacteria, but we found that a little damage induced by the inoculation, caused that *S. pyogenes* remained an average of 107 h instead of 56 h recorded in non-injured pinna. We found that 100% of mice inoculated by *S. pyogenes* without damage could clear the bacterium in 96 h, while 100% of mice inoculated in lesions delayed until 192 h to eliminate it, the difference was statistically significant (*p* <0.00001, Wilcoxon test) (Fig. 6).

These results show that *S. pyogenes* can remain in the model of infection, and could be easily detected by a standard culture from the infection site.

### Best conditions for the model of dermal infection

One of the main aims of this study was to develop a quantitative model of dermal infection, the aim was to discriminate among different levels of severity in the infection and inflammation; we tested 2 areas of inoculation to find the best one for developing the model. Both selected areas were localized on the auricle, because of its flat shape, that characteristic let us to obtain more precise measures.

**Fig. 7:**
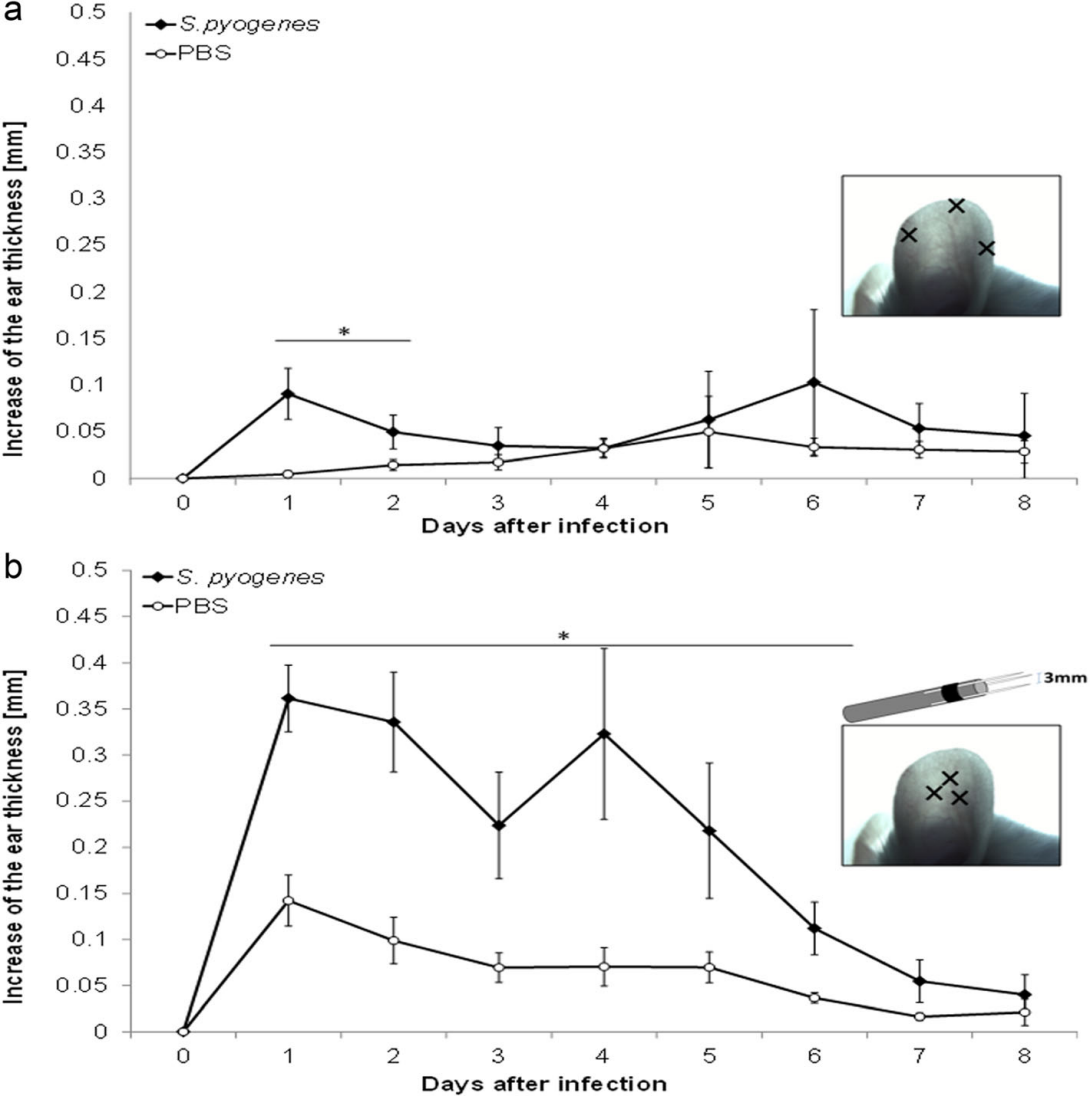
Best area for inoculation. We used two ways to inoculate *S. pyogenes* on the mouse pinna, in both methods the pinna was pierced with 23-gauge hypodermic needles over three points; the graphs show the average between the increases of the ear thickness caused by the damage on the pinna and recorded every day. *S. pyogenes* was inoculated in one pinna while the contralateral was inoculated with PBS as control, the deviation bars show SEM. **a** Inoculation near to the edge of the auricle: we found statistical difference in ear thickness during the first 2 days after the inoculation (*n* = 8, *p* <0.047 paired t student test). **b** Inoculation at the center of the auricle with a punch made with 3 needles arranged in a triangular shape: we found a statistical difference in ear thickness during 6 days post-inoculation, (*n* = 8, *p* <0.048 paired t student test)

When the infection was performed at the marginal edge of the auricle, the increase of the ear thickness induced was statistically different to the PBS-treated control, but only the first 2 days post-inoculation (*p* <0.047, Fig. 7a). On the other hand, when the center of the auricle was infected, the oedema induced was bigger and there was a statistic difference versus PBS-treated control during the first 6 days post-inoculation (*p* <0.048, Fig. 7b). All those results were the base to choose the second area for developing the model.

**Fig. 8:**
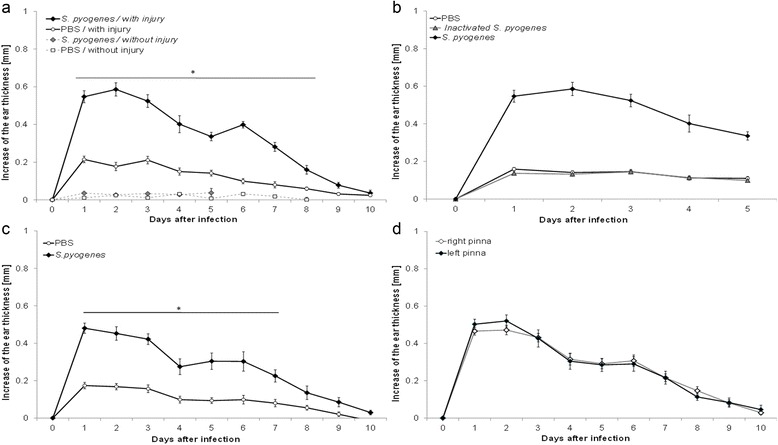
Experimental conditions for the model. The increase of the ear thickness (oedema) after inoculation was evaluated considering some experimental conditions. The statistical analysis was performed comparing daily the increase of the ear thickness, induced by *S. pyogenes* in contrast with the PBS control, the deviation bar shows the SEM. **a** Two independent groups were tested, in both, a little damage was performed at the mouse pinna, we found statistical difference (*) during 8 days post inoculation (*n* = 8, *p* <0.0002, non-paired t student test) between *S. pyogenes* and PBS-control. We evaluated 2 independent groups but without injury, and we did not find difference in the ear thickness between mice inoculated with *S. pyogenes* or PBS (*n* = 5). **b** Two independent groups were compared: mice inoculated with *S. pyogenes* viable and mice inoculated with *S. pyogenes* inactivated, both in injured pinna. The inactivated bacteria failed to induce the oedema, and when it was compared with PBS-treated control there was not difference (*n* = 4 *p* > 0.15, non-paired t student test). **c** One group was inoculated in both damaged pinna, one of them were infected with *S. pyogenes* and the contralateral was de control, treated with PBS, we found statistical difference during 7 days post-inoculation (*n* = 14, *p* < 0.0046 paired t student test). **d** There was no difference between the oedema caused by *S. pyogenes* in the right or left pinna (*n* = 8, *p* > 0.12, paired t student test)

The increase of the ear thickness induced by the *S. pyogenes*, was tested in mice without any damage, the inoculation on the auricle did not cause any change in the ear thickness, only when the bacterium was inoculated on a previous lesion, we observed a statistically significant increase of the ear thickness, (*p* <0.0002, Fig. 8a).

*S. pyogenes* apparently can induce an increase of the ear thickness, however that increase could be caused by the antigenic proprieties of the bacterium instead of the infection process. We carried out inactivation of *S. pyogenes* by 3 cycles of freezing and thawing of a suspension with 1 × 10^10^ CFU/ml in PBS, four mice with damaged auricles as previously described above, were treated by using this inoculum, we confirmed the inactivation process by culturing 10 μl of the suspension. During the next 5 days after inoculation, we measured the ear thickness; there was no difference with the PBS-treated control group (*p* > 0.15, Fig. 8b). These results confirm that *S. pyogenes* can induce a dermal infection and is the cause of the oedema on the inoculation site.

### Strength and precision

Both auricles from the same animal were inoculated with *S. pyogenes* at 1 × 10^8^ CFU or PBS (*n* = 14); the process was carried out in a blind way. Every day during 10 days the increase of the ear thickness was measured, and we found statistic difference during the first 7 days post-infection between the infected pinna and the control pinna (*p* <0.0046, Fig. 8c). On the other hand, the magnitude of the oedema caused by *S. pyogenes* was compared; when the bacterium was inoculated into the left or right pinna from eight mice, there was no difference between the left or right pinna during all experiments (*p* > 0.12, Fig. 8d). We assessed the precision of the model by considering the standard deviation and variation coefficient of the increase of ear thickness; results are shown in Table 1.

**Table 1:** Precision in the measure of the increase of the ear thickness

	Days after inoculation									
	1	2	3	4	5	6	7	8	9	10
Mean (mm)	0.547	0.586	0.524	0.402	0.336	0.393	0.281	0.149	0.063	0.036
Median (mm)	0.553	0.666	0.529	0.357	0.315	0.389	0.252	0.116	0.062	0.022
SD	0.128	0.140	0.134	0.180	0.089	0.065	0.095	0.081	0.043	0.062
V^a^	23.424	23.935	25.530	44.916	26.488	16.534	33.630	54.383	79.277	170.659

^a^ V = Coefficient of variation, expressed as percentage

### Validation of model of dermal infection

**Fig. 9:**
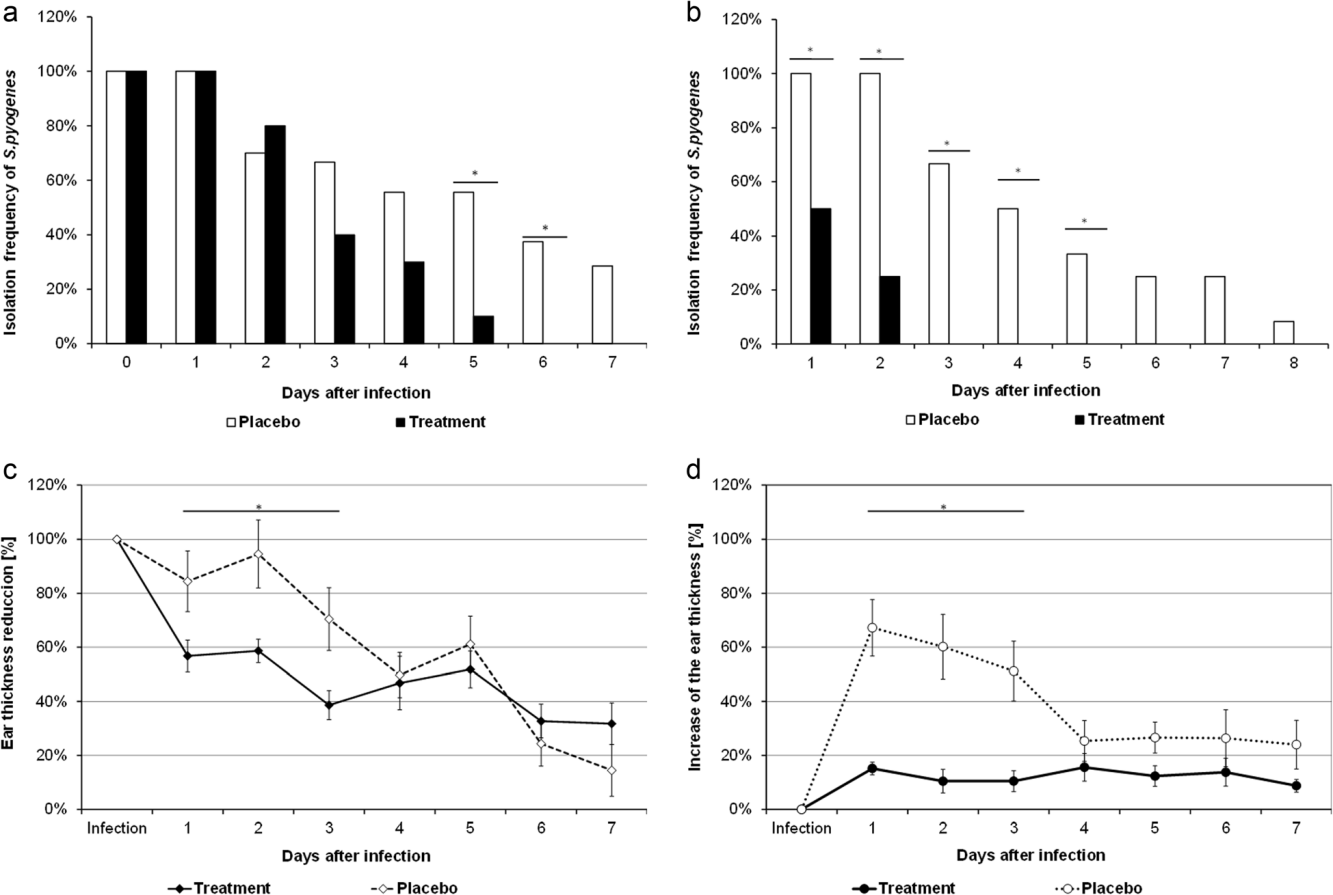
Effect of a standard treatment on the model of dermal infection and oedema. We analyze the effect of an otic formulation made with Ciprofloxacin as antibacterial and Hydrocortisone, Benzocaine as anti-inflammatory treatment on a dermal infection induced by *S. pyogenes*, The bacterium was inoculated in both pinna of 12 mice as previously mentioned, and the treatment was assessment in comparison with a placebo. Two kind of application were evaluated: as a curative (24 h post inoculation) or preventive (1 h post-inoculation). Treatments were applied in a blind way but considering that one infected pinna was exposed to the standard treatment and the contralateral was treated with a placebo (glycerol 50%). **a** Antibacterial effect of curative treatment applied 24 h post inoculation, *S. pyogenes* could resist the first 5 days of the treatment with the ciprofloxacin in the standard formulation, we found statistical difference only from the 5 to 6 day of treatment in comparison with placebo (*p* <0.034, *x* ^2^ test). **b** Antibacterial effect of preventive treatment, when the standard formulation was applied 1 h post inoculation, we could observe that *S. pyogenes* was not able to survive more than 2 days in presence of the antibiotic, we found statistical significant difference during the first 5 days (*p* <0.0285, *x* ^2^ test). **c** Anti-inflammatory effect of the curative treatment. 24 h after inoculation, *S. pyogenes* caused an increase of the ear thickness that represent 100%, when applied the formulation with hydrocortisone as standard anti-inflammatory drug, we found a decrease in the ear thickness that was statistically significant with the placebo during the first 3 days (*p* <0.026, paired t student test). **d** Anti-inflammatory effect of the preventive treatment. 1 h after inoculation of *S. pyogenes*, the pinna were treated with the formulation, we found that standard anti-inflammatory drugs was able to prevent the increase in thickness observed in placebo-treated pinna, the difference was significant during the first 3 days (*p* <0.0032, paired t student test)

A robust model of dermal infection should present not only accuracy and reproducibility, but also it should display expected results with standard anti-inflammatory or anti-microbial drugs. We carried out a protocol where we infected 12 mice following the consideration above mentioned, when we applied the antibacterial formulation that contain ciprofloxacin. In a curative way we can observe that *S. pyogenes* is able to remain into the infected pinna during 5 days, only 2 days less than placebo (Fig. 9a) in contrast when the treatment was administered as a preventive way, just 1 h post-inoculation, the bacterium was only able to resist 2 day the antibacterial effect (Fig. 9b) In both administration ways *S. pyogenes* were eliminated from the infection site before the infected pinna treated with placebo. We found that otic preparation which contain Hydrocortisone, presented an important anti-inflammatory effect when it was administered in both ways tested here: curative (Fig. 9c) and preventive (Fig. 9d) the differences when are compared with the placebo are notorious during the first 3 days post-inoculation, however when the treatment was applied in a preventive way, we noticed that practically prevented the increase in the ear thickness, as compared to that observed with the placebo-treated pinna.

## Discussion

Preclinical assays should be tested in models that emulate the common signs found in the disease. In the case of dermal infections, a useful model should show the inflammatory process and also the presence of infectious agent. Most animal models also display differences in their susceptibility to several microorganisms. *S. pyogenes,* a recognized human pathogen [21] was selected as infectious agent, as some strains are able to infect skin [22, 23] and are responsible for cases of erysipelas or impetigo [24, 25]. On the other hand, there are evidence that *S. pyogenes* can also infect BALB/c mice in a previous injury on the dermis [16]. Our strain was previously isolated from a patient with impetigo, where we isolated a strain that can infect mice skin, based on improved fitness for in vivo growth. Even though we are not aware of how many genetic mutations might improve such phenotype at a population level, or how stable over serial subcultures this phenotype is, we achieved an increase of the ear thickness at least 2 times bigger than non-inoculated control, (52 of 54 test in a total of 5 independent experiments), in more than 95% of the mice infected with *S. pyogenes* B-50879. Taking into account the above, we consider that our strain is stable and therefore is available in ARS Culture and Patent Culture Collections. Therefore, we consider that our strain has the requirements for using it as infectious agent in the present model.

The adaptation to different mice breed would be worth assessing, although for this particular study it was out of our scope. As it is known that the skin harbors many immune cells (Langerhans cells; keratinocytes; dendritic epidermal T lymphocytes; epidermotropic lymphocytes; and melanocytes, epidermal pigment cells with immune properties) [26] as well as B cells [27], some heterogenicity in host response might exist.

Although some strains of *S. pyogenes* can infect skin, it is necessary that a previous lesion or damage in the epithelium exists, so once the bacterium has infected it, these can spread to other areas [2, 3, 24, 28]. When *S. pyogenes* was inoculated in mouse pinna without a previous lesion, the bacterium did not infect the auricle, and there was not an evident change in the mouse pinna. Those results show the importance of the previous lesion to achieve the infection, and agree with previous works where is also necessary to induce a lesion before inoculation [19, 29, 30].

Human cutaneous infections induced by *S. pyogenes* frequently develop abscess, erythema and crusts, also there are leukocytes released in the exudate from those lesions [2, 3, 24]. In BALB/c mice infected by the strain *S. pyogenes* B-50879, we observed similar signs and coccid bacteria into the infected tissue. In a mice model of infection in the dorsal area, it has been recorded that *S. pyogenes* can be maintained in the animal 3 to 5 days post-inoculation [16, 18]. Our model showed similar results, where 108 h was the average time in where we could recover the bacterium from the infection site, although in some cases the bacterium was recovered up to 8 days post-inoculation. We consider that this time is enough for assessing the antibacterial effect of any treatment.

Tissues infected by the strain *S. pyogenes* B-50879 showed an increase of the keratinized squamous epithelium stratum, oedema and infiltration of polymorphonuclear leukocytes into the connective tissue. These data agree with an infection model of external otitis, developed in rats infected with *Pseudomonas aeruginosa* or *Candida albicans* [15]. In order to verify that an infection process instead of an inflammatory response stimulated by bacterial antigens caused our results, we inoculated the same bacterial load but as non-viable microorganisms, the results showed similar findings as the PBS-treated control. Those results led us to consider that an infection process produced by *S. pyogenes* in fact induced the inflammatory response we observed.

All injury into the skin involves tissue damage that might induce an inflammatory response. A previous model was developed by a mechanical lesion into the external ear canal [31]. Our model requires a previous injury at the pinna, which in fact induces an increase of the ear thickness, however it is statistically lower than the oedema induced by the *S. pyogenes* inoculation. That difference is observed at least 7-days post-inoculation, a period of time that might be useful for testing the anti-inflammatory efficacy of treatments.

Infectious diseases can induce a release of polymorphonuclear leukocytes into the infected tissues; we observed an increase of these cells in peripheral blood as well as tissues inoculated with the bacterium. Considering all results presented here we conclude that *S. pyogenes* B-50879 is able to cause a localized infection in a mice pinna, that induce an oedema similar to other models, and that furthermore can emulate the typical signs found in human dermal infections.

Although there are previous models of dermal infection in mice by using *S. pyogenes* [16, 18, 19], those are not quantitative models, because the infection is induced in anatomical areas that are not easily accessible to obtain measures of eodema, and the inflammatory diagnosis is based in histopathological resources, that might be susceptible to ambiguous results in mice with different severity of disease.

Analytical methods require objective measures; this is not an exception from animal models developed to assess the anti-inflammatory effect of molecules. However, many of them require histopathological methods [15], which could increase the cost and time of process because it would be indispensable that interpretation is carried out by a pathologist. However, it is likely that two results from the same sample do not display similar results, because the interpretation is based in personal criteria. Previously, there have been developed several quantitative models of inflammation considering: the measure of the ear thickness by using a micrometer [4, 32, 33]; the weight of biopsies from mice’s auricle, previously stimulated by chemicals [11, 34, 35]; or even through the microscopic measure of the tissue thickness from histopathological slides [8]. The model of dermal infection described in this work considered the anatomical advantage of mouse pinna because it has a flat shape, that let us to obtain precise measures of the ear thickness, directly related with the oedema. The main difference with previous models is that a bacterium is the source that causes infection and inflammation instead of chemicals that can induce oedema but not infection, limiting its use for assessing anti-inflammatories proprieties [4, 9, 11]. Our model emulates a dermal infection, which is characterized by a localized lesion, erythema, exudate and oedema, we confirmed that results with conventional histopathological methods, in where we observed inflammatory cells, also there was an increase in the number of peripheral blood neutrophils, specific leukocytes specialized to clear bacterial infections. In order to determine if the model developed is useful for testing anti-inflammatory and antibacterial formulations, we carried out a protocol where we tested the antibacterial effect of ciprofloxacin, antibiotic that has been previously tested as otic formulations [36, 37], and anti-inflammatory effect of hydrocortisone, which has been used as standard of anti-inflammatory effect in skin [4]. Our results showed that the model is useful when the drugs are tested in a curative way as well as preventive way. We consider that the method also can be useful for testing anti-inflammatory treatments, because our results showed the expected anti-inflammatory response in the pinna treated with the hydrocortisone contained in the formulation, the results presented here, were similar to the oedema inhibition after the treatment with the anti-inflammatory treatment, in a model of oedema stimulated by TPA [4, 9]. However, we consider that our model has an advantage, because in fact it is emulating the dermic inflammation caused by the infection.

All findings agree with the hypothesis that *S. pyogenes* B-50879 is able to cause an infection in the mouse pinna in where it is possible to track the presence of the pathogen, evaluate consequences of infection such as oedema through the measure of the ear thickness, as well as to test new potential treatments with antibacterial or anti-inflammatory effect.

## Conclusions

The best method tested for developing a precise oedema was to inoculate *S. pyogenes* in a previous lesion in the center of auricle. The increase of the ear thickness was consistent and in all experiments, we could observe an increase close to 3 times the original size of the auricle, instead of 1.5 observed for the PBS-treated control; this difference was statistically significant. The magnitude of the oedema induced by our method was similar to those induced by TPA [4, 9], croton oil [38] and was bigger to the one induced by mustard oil [33, 39] or capsaicin [13]. The difference was statistically significant during 7-days post-inoculation and the precision observed was adequate to differentiate auricles inoculated with controls from those infected by *S. pyogenes*. Similar results were found when the experiment was conducted in paired design (comparing left and right auricles from the same animal) or non-paired design (when were compared different groups of animal such as control group or infected group), even when the experiment was performed in a blind way. These results support the conclusion that our model is strong and robust, and we recommend to use this animal model in randomized designs performed in a blind way. The method is also useful for testing molecules or treatments with anti-inflammatory or antibacterial proprieties.

## Abbreviations

MEM-FBS: Minimal essential medium supplemented with 10% fetal bovine serum
PBS: Phosphate buffer saline pH 7.2
PIR: Pirrolidonyl arylamidase
SEM: Standard error of the media
TPA: 12-O-tetradecanoylphorbol-13-acetate
